# The Role of Constitutional Copy Number Variants in Breast Cancer

**DOI:** 10.3390/microarrays4030407

**Published:** 2015-09-08

**Authors:** Logan C. Walker, George A.R. Wiggins, John F. Pearson

**Affiliations:** 1 Mackenzie Cancer Research Group, Department of Pathology, University of Otago, Christchurch 8140, New Zealand; E-Mail: george.wiggins@otago.ac.nz; 2 Biostatistics and Computational Biology Unit, University of Otago, Christchurch 8140, New Zealand; E-Mail: john.pearson@otago.ac.nz

**Keywords:** copy number variants (CNVs), breast cancer, SNP arrays, risk, genetic variation

## Abstract

Constitutional copy number variants (CNVs) include inherited and *de novo* deviations from a diploid state at a defined genomic region. These variants contribute significantly to genetic variation and disease in humans, including breast cancer susceptibility. Identification of genetic risk factors for breast cancer in recent years has been dominated by the use of genome-wide technologies, such as single nucleotide polymorphism (SNP)-arrays, with a significant focus on single nucleotide variants. To date, these large datasets have been underutilised for generating genome-wide CNV profiles despite offering a massive resource for assessing the contribution of these structural variants to breast cancer risk. Technical challenges remain in determining the location and distribution of CNVs across the human genome due to the accuracy of computational prediction algorithms and resolution of the array data. Moreover, better methods are required for interpreting the functional effect of newly discovered CNVs. In this review, we explore current and future application of SNP array technology to assess rare and common CNVs in association with breast cancer risk in humans.

## 1. Introduction

Over the past decade there have been a large number of studies that have explored the biological impact of constitutional (inherited and *de novo*) copy number variants (CNVs) in the human genome [1,2]. CNVs are structural rearrangements that increase or decrease DNA content at regions larger than 50 base pairs (bps) in size [1,2], accounting for a majority of genetic variation in humans based on bp coverage. These variants are estimated to cover 5%–10% [2] of the human genome which is at least an order of magnitude greater than the number of bps (~15 Mbps; dbSNP Human Build 142) encompassed by the more commonly studied single nucleotide polymorphisms (SNPs).

Molecular technologies used to profile DNA copy number, such as microarrays (SNP-based arrays and comparative genomic hybridisation) and next-generation sequencing, have led to the identification of more than 300,000 CNVs, or 21,757 unique CNV loci in the human genome [3] . These technologies have also revealed the extent to which constitutional CNVs partially overlap or fully encompass genes and/or regulatory sequences. Concomitant gene expression analyses have shown a strong relationship between copy number dosage and mRNA levels with hundreds of genes [4,5]. This functional effect can play an important role in a variety of human diseases, including breast cancer [6,7,8,9].

## 2. Single Nucleotide Polymorphism (SNP)-Array Platforms to Assess Breast Cancer Risk

A significant proportion of breast cancers arise in a subset of women who have multiple affected relatives as a result of inherited genetic factors that increase the risk of developing the disease. The relative risk (RR) of breast cancer in mothers and sisters of patients is increased, ranging from 1.8-fold to more than 5-fold [10,11]. In 5%–10% patients, inherited mutations in highly penetrant cancer susceptibility genes, such as *BRCA1* and *BRCA2*, are known to confer a significantly elevated risk (>10-fold) of breast cancer and their carrier relatives [12]. A further 5% of cases carry deleterious variants in moderate-risk breast cancer susceptibility genes, such as *CHEK2*, *ATM*, *BRIP1*, and *PALB2* [11,12,13,14]. However, these variants are too rare to be identified in most genome-wide association studies and do not increase risk sufficiently for capture by linkage analysis in family studies.

Numerous genome-wide association studies for different population groups have successfully been performed to discover low-risk SNP variants that are associated with breast cancer [15,16,17,18,19,20,21,22,23,24,25,26,27,28,29,30,31,32,33]. Such studies have been underpinned by SNP array platforms from companies, such as Affymetrix, Illumina and Perlegen Sciences, ranging in genome coverage, spatial resolution and design. Probes used on SNP arrays for these studies have generally been selected to target SNPs with a minor allele frequency greater than 5%. Thus, genome-wide association studies are designed to detect causal variants that are relatively common in the population. As breast cancer studies have grown in size, less common variants are able to be assessed for risk association. A recent initiative as part of the Collaborative Oncological Gene-Environment Study (COGS) used a custom-designed array to assess almost 200,000 SNPs across the genome in approximately 50,000 breast cancer cases and 50,000 controls [28]. Studies of this size are statistically powered to evaluate variants with a minor allele frequency <5%. As a result of the large COGS initiative, more than 90 independent common susceptibility loci have now been identified, explaining a further 16% of the familial risk [27].

Currently known low-, moderate- and high-risk genetic factors explain up to half of the familial clustering in breast cancer [28]; thus, for a substantial fraction of women, the genetic changes contributing to breast cancer remains undetermined, even if they have a family history [34]. Discovery of variants to explain this “missing heritability” is of clinical relevance, but will require different approaches that perhaps include other types of genetic variation, such as CNVs, using high throughput technology. 

## 3. Copy Number Variant (CNV) Prediction Algorithms for SNP Array Data

The ability to study CNVs at a genome-wide level has been made possible by the development of high-throughput SNP array technologies. Moreover, the vast amount of SNP-genotyping data generated by numerous genome-wide association studies of breast cancer offers significant potential to explore the contribution of CNVs to this disease. SNP markers present on many early Affymetrix and Illumina arrays were also supplemented with thousands of intensity-only (non-polymorphic) probes that target known CNV regions, especially those regions unsuitable for SNP genotyping probes.

A large number of CNV calling algorithms have been applied to SNP array and/or array comparative hybridisation data in published studies with variable success. A proportion of these algorithms have been utilised more frequently for a variety of reasons, including accuracy, availability and suitability to the array platform used in the studies and ease of implementation. Most algorithms are either proprietary and available commercially, or have coded implementations freely available for downloading. Table 1 lists those in common use by the citations of their principal publication in PubMed at the time of writing. A measure we acknowledge underestimates the popularity of commercial (and usually unpublished) solutions.

### ACCURACY of CNV Predictions from SNP Arrays

A major limitation for the use of SNP arrays in CNV association studies is the accuracy of CNV calling algorithms. The current CNV algorithms vary in methodology and subsequently produce varied results (Table 1). The most numerous CNV calling methods use Hidden Markov Models (HMM) to estimate copy number at loci with transition probabilities estimated or supplied, as for example from gold standard datasets. Others methods use mixtures—particularly Gaussian—distributions, or Bayesian methods. Many implementations include heuristics to deal with or explicitly model features in the data such as loss of heterozygosity regions and GC waves, and set a minimum number of probes for which they will call a CNV.

Methods have been proposed that might reduce false positives, including altering parameters within the algorithms (*e.g*. CNV size and number of probes included) and comparing multiple algorithms [35]. Validation of predicted structural variants is critical for the use in association studies. Table 2 provides a list of studies that explored the issue of algorithm accuracy. Three studies [36,37,38] assessed the accuracy of multiple CNV calling algorithms by comparing data they derived from samples previously used in “gold standard” studies [39,40]. These reports present different conclusions with respect to algorithm performance, although PennCNV was the only algorithm included in all three studies. Winchester and colleagues validated 49% of CNVs predicted by PennCNV in the Kidd *et al.* [40] study for the highest rate in their study. Zhang and colleagues used multiple permutations to obtain the greatest recovery of CNVs from gold standard studies using the same samples. For PennCNV with pedigree information included, a maximum recovery rate (number of CNVs in Conrad *et al.* [39] that were predicted) was only 35% using >20 probes. Birdsuite was able to recover nearly half of the predicted CNVs (48%) under similar setting (no pedigree information). Zhang *et al* [38] found deletions were validated at a much higher rate with both Partek and Birdsuite correctly predicting deletions selected for validation (5/5). In comparison, predicted duplications showed a high false positive rate with PennCNV, the most accurate predicting 66.7% of CNVs validated (4/6) [38]. Similarly, Seiser and Innocenti assessed three samples previously characterised in Conrad *et al.* [39] to measure the performance of three HMM algorithms (GenoCN, PennCNV and QuantiSNP) [36]. PennCNV performed poorly with low sensitivity (14.46%, minimum of five probes) and high specificity (a common trait for HMM algorithms). With exception of Zhang *et al.* [38], many studies were limited by the reliance on CNVs from previously published reports as there was no attempt to experimentally validate predicted variants. Zhang and colleagues illustrate this vulnerability by highlighting disagreement with commonly used gold standards from Conrad *et al.* [39] and Kidd *et al.* [40]. Comparing CNVs calls in five samples used by each study showing strikingly poor agreement [38]. Other studies have used mass spectrometry, quantitative polymerase chain reaction (qPCR) and/or multiplex ligation-dependent probe amplification (MLPA) to attempt to validate CNVs [41,42]. Typically, these studies used methods to reduce false positives by creating strict criteria for inclusion. One study confirmed that sensitivity was a weakness of CNVPartition, PennCNV and QuantiSNP, with QuantiSNP showing the greatest MLPA-validated sensitivity (28%) [42]. This study also showed that, of the true positives, each algorithm tended to correctly predict the CNV class (homozygous deletion, heterozygous deletion and duplication) with sensitivity >92% and specificity >87%. An exception to these results was the ability of QuantiSNP to accurately call homozygous and heterozygous deletions, with call rates of 68% and 62%, respectively). Together, these studies highlight the lack of a consensus on CNV-calling methodologies used to assess SNP array data. Furthermore, results from publications reviewed in Table 1 support the necessity to experimentally validate any CNV loci that are predicted by SNP array data, and are to be included in breast cancer association studies

**Table 1:** Commonly (>10 citations) applied CNV detection methods for SNP-array data.

Software	Algorithm	Code	Platform	Year ^a^	Reference	Citations ^b^	Software URL
PennCNV	HMM	Perl	Multiple	2007	[43]	300	http://penncnv.openbioinformatics.org
Birdsuite (Birdseye, Canary)	Mixture models	Java/Python/R	Affymetrix	2008	[44]	300	http://www.broadinstitute.org
Nexus Copy Number	Proprietary (Segmentation)	windows executable	Multiple	-	-	100	http://www.biodiscovery.com
QuantiSNP	HMM	MATLAB	Multiple	2007	[45]	100	http://sites.google.com/site/quantisnp
CNVPartition	Proprietary	windows executable	Illumina	2006	-	100	http://support.illumina.com
Partek Genomics Suite	Proprietary (Segmentation or HMM)	windows executable	Multiple	-	-	30	http://www.partek.com/pgs
CNVFinder	Experimental variability	perl	Array CGH	2006	[46]	30	http://www.sanger.ac.uk/resources/software/cnvfinder/
CGHCall	segmentation and mixture model	R	Array CGH	2007	[47]	30	http://www.few.vu.nl/~mavdwiel/CGHcall.html
GenoCNV	HMM	R	Multiple	2009	[48]	30	http://www.bios.unc.edu/~weisun/software/genoCN.htm
SW-ARRAY	Smith Waterman	R	Array CGH	2005	[49]	30	Not available
HMMSeg	HMM wavelet smoothing	Java	Multiple	2007	[50]	10	http://noble.gs.washington.edu/proj/hmmseg
VanillaICE	HMM	R	Affymetrix	2008	[51]	10	http://cran.r-project.org
CNVHap	HMM, Haplotype	Java	Multiple	2010	[52]	10	http://www.imperial.ac.uk/people/l.coin
dChip	Multiple	R	Multiple	2008	[53]	10	http://sites.google.com/site/dchipsoft
GADA	Bayesian	R	Multiple	2010	[54]	10	http://cran.r-project.org
CNV Workshop	Segmentation	complete VM	Multiple	2010	[55]	10	http://sourceforge.net/projects/cnv

^a^ Year reference when published. ^b^ At least this many citations in PubMed or company website at July 2015. Abbreviation: HMM, Hidden Markov Model.

**Table 2:** Accuracy of CNV-calling algorithms.

Algorithm(s)	Platform	Validation Method	Accuracy	Study Conclusion	Reference
Adapted method on SW-ARRAY and GIM	Affymetrix	qPCR or Mass Spec Validation	2.5% false positives, ~90% singleton validation	Developed a multistep algorithm to better call CNVs.	[41]
Birdsuite, CNAT, CNVPartition, GADA, Nexus, PennCNV and QuantiSNP	Affymetrix, Illumina	Comparison of HapMap samples to Kidd *et al.*, Korbel *et al.* and Redon *et al.*, data [5,40,56]	Assay sensitivity ranged 20%−49% with some algorithms predicting more events (*i.e*., GADA, 546 predicted CNVs).	PennCNV had the greatest sensitivity (49%). Little agreement between studies and within studies.	[37]
cnvHap, CNVPartition, PennCNV and QuantiSNP	Aglient, Illumnina	Compared samples either with previously characterized (by aCGH) CNVs or HapMap samples from Kidd *et al*. [40]	cnvHap had very good sensitivity (68%) for larger CNVs (>10kb) in Kidd *et al.* This reduced to 31% for smaller CNVs (<5kb).	cnvHap has increased sensitivity compared with other CNV algorithms.	[52]
PennCNV, Aroma.Affymetrix, APT and CRLMM	Affymetrix	Compared concordance between calling algorithms.	Greater concordance in deletion (51.5%) than duplications (47.9%). The probable false positive rates for CRLMM and PennCNV were 26% and 24%.	PennCNV appeared to detect all the CNV and more than CRLMM predicted	[57]
CNVPartition, PennCNV and QuantiSNP	Illumnina	Agreement between algorithms	Agreement varied from 59%−62% for deletions, to 43%−57% for duplications.	Use of multiple algorithms increased the positive predictive value, as did the number of probes and the minimum size (kb).	[35]
CNVPartition, PennCNV and QuantiSNP	Illumnina	MLPA validation, measures were taken to reduce false positive calls.	All algorithms show better specificity than sensitivity. QuantiSNP was the most sensitive, predicting 28% of CNVs. PennCNV was better at discriminating copy number state.	Applying methods to reduce false positives results in low sensitivity.	[42]
ADM-2, Birdsuite, CNVfinder, CNVPartition, dCHIP, GTC, iPattern, Nexus, Partek, PennCNV, QuantiSNP	CGH arrays and SNP arrays (Affymetrix and Illumina)	Experiments were repeated in triplicate and CNV calls were compared. CNV calls were also compared to 5 references (‘gold standards’).	Algorithm replication has <70% reproducibility. CNV calls between any two algorithms is typically low (25%–50%) within a platform. Overlap with DGV was high, whereas overlap with references [39,40] was low.	Newer high resolution arrays outperform older arrays in both CNVs’ call and reproducibility. Algorithms developed for specific array platforms outperformed adapted and independent algorithms.	[58]
Birdsuite, Partek, Genomics Suite, HelixTree and PennCNV	Affymetrix	Comparison with HapMap CNV in two studies [39,40].	Overlap ranged between 42% and 70% when including 20 probes for Kidd *et al.* [40] and 26%−48% in Conrad *et al*. [39]	Birdsuite outperformed the other 3 algorithms over multiple permutation.	[38]
		qPCR validation of rare CNVs (a single CNV event in >1000 bipolar samples)	For each algorithm between 10 or 11, CNVs were tested. Partek and Birdsuite both validated all (5/5) deletion events tested.	Birduite and Partek had high positive predictive values, particularly with deletions. HelixTree performed poorly.	
CNVPartition, PennCNV and QuantiSNP	Illumnina	Comparison to a previous CGH study [59]. qPCR validation of 3 candidate loci in 717 horses.	50 CNVs were called by all 3 algorithms. QuantiSNP had the highest overlap with CNVs predicted from CGH arrays (25%). Validation rates were greater than 80% for the 3 loci.	CNVPartition predicted the least CNVs, suggesting a high false negative rate.	[60]
GenoCN, PennCNV and QuantiSNP	Illumnina	Comparison of HapMap sample to Conrad *et al.*[39] Compared both CNVs (*i.e*. Gain or Loss) and normal calls.	All algorithms show much better specificity than sensitivity. PennCNV had the worst sensitivity, predicting <15% of Conrad *et al.* [39] CNVs in 3 samples	The three HMM algorithms all performed with varied results. They were all highly specific (>98%), but sensitivity remains to be an issue for all three algorithms.	[36]
cnvHap, COKGEN, GenoCNV, HaplotypeCN, PennCNV and QuantiSNP	Affymetrix	Compared 270 HapMap samples which have been previously described. Compared simulated data to test haplotype phasing between cnvHap and HaplotypeCNV.	GenoCNV has the most sensitivity (28%) when using Kidd *et al.* [40]; however, the concordance rate in PennCNV was greater (36% and 9%, respectively).	Algorithm performance varied with reference study. GenoCNV was the most sensitive but had the lowest concordance rate. HaplotypeCNV, cnvHap and PennCNV (under a specific permutation) were compared separately, with HaplotypeCN outperforming the other two.	[61]
Birdsuite, dCHIP, GTC and PennCNV	Affymetrix	Comparison to a previous CGH study [62].	GTC had the highest portion of CNV matching (50% overlap) to CGH, 66%. Larger CNVs were called with greater accuracy.	Birdsuite called the most CNVs; however, PennCNV outperformed all algorithms with greater specificity and sensitivity.	[63]

Abbreviations: aCGH, array comparative genomic hybridisation; APT, Affymetrix Power Tools; CNV, copy number variant; CRLMM, corrected robust linear mixture model; DGV, Database of Genomic Variants (http://dgv.tcag.ca/dgv/app/home ); HMM, hidden Markov model; GTC, Genotyping Console; kb, kilobases; MLPA, Multiplex ligation-dependent probe amplification; qPCR, quantitative polymerase chain reaction.

## 4. Functional Annotation of CNVs

The functional impact of CNVs in the human genome vary as a result of the variant size, copy number state, and location relative to genes or key regulatory regions. Homozygous deletions overlapping at least 85% of exons from approximately 100 protein-coding genes have been identified in genomes from seemingly healthy individuals [2], suggesting these genes are functionally redundant or are related to an unknown phenotype. Haploinsufficiency for genes disrupted by a hemizygous deletion is also an important mechanism for genetic disease, such as *APOBEC3B* and breast cancer risk [6,64]. Conversely, gene duplications resulting from overlapping CNVs can influence biology through triplosensitivity.

There is an increasing number of CNVs of unknown clinical significance that are predicted to be involved in disease susceptibility due to potentially deleterious effects on overlapping or nearby gene(s). Despite the myriad of computational tools developed to detect CNVs for different array and sequencing platforms, a significant informatics challenge exists for interpreting both the functional and clinical role of these variants. Computational tools, such as SG-ADVISER CNV [65], CNV-WebStore [66] and CNVannotator [67], have been developed to derive functional effects from predicted variants. These tools are useful for assigning potential clinical implications of CNVs based on their location within known pathogenic regions. To assess variant pathogenicity, SG-ADVISER CNV utilises additional factors to generate a classification score, including 1) allele frequency information from repositories, such as the 1000 Genomes Project; and 2) clinical genetic information from databases, such as Online Mendelian Inheritance in Man [68], ClinVar [69]. However, a major limitation of annotating CNV regions derived using SNP arrays is the inability to precisely define their breakpoints. Thus, any overlap between predicted CNVs with clinically relevant regions along the genome remain putative without further validation using ancillary techniques, such as quantitative PCR or MLPA.

## 5. Application of SNP Arrays for Profiling CNVs in Breast Cancer

Structural variants, including CNVs, contribute to many complex diseases, and could account for some of the missing heritability of breast cancer. CNVs have been reported to encompass genes known to be involved in breast cancer susceptibility, including *BRCA1* and *BRCA2,* and therefore may similarly affect other genes involved in breast cancer-related pathways [12].

### 5.1. Inherited Copy Number Polymorphisms and Breast Cancer Risk

Analysis of large genome-wide association studies carried out by the Wellcome Trust Case Control Consortium suggested that common CNVs were unlikely to play a major role in breast cancer susceptibility [70]. This study used a 105K probe Agilent CGH array design containing probes tagging for copy number loci previously identified from (1) the Genome Structural Variation (GSV) Consortium [39]; (2) CNV studies using the SNP arrays Affymetrix 6.0, Illumina 1M, and Affymetrix 500k; (3) novel sequence absent from the reference sequence; 4) candidate genes; and 5) additional risk-associated loci. However, this study was not sufficiently powered to detect the effects of low-penetrant alleles with a minor allele frequency (MAF) less than 5%. Moreover, the genomic regions assessed by this study were limited by the design of the arrays used to generate genotype information across the genome. More recently, a genome-wide association study of common CNVs (MAF ≥ 5%) conducted among Chinese women using high-resolution data from the Affymetrix SNP Array 6.0 identified a deletion in the *APOBEC3* gene cluster associated with breast cancer risk. Within this population, the deletion was identified in 65% cases and 45% of controls, conferring odds ratios (ORs) of 1.3 and 1.8 for a hemizygous and homozygous deletion, respectively (*p* = 2.0 × 10^−24^) [6]. Subsequent investigations of women with European ancestry using quantitative-PCR also observed the deletion, albeit at a much lower population frequency [71]. Comparable to the study of Chinese women, a higher proportion of breast cancer affected European women (12.4% *vs.* 10.4%, respectively) because they carried the *APOBEC3* allele, thereby conferring low to moderate risk of disease (ORs of 1.2 and 2.3 (*p* = 0.005) for a hemizygous and homozygous deletion, respectively). Interestingly, the same deletion (CNV ID: CNVR8164.1) was originally identified by the Wellcome Trust Case Control Consortium; however, replication experiments did not show a significant association with breast cancer.

As mentioned above, there is now a wealth of array data available from SNP-based genome-wide association studies that can be utilised for assessing the contribution of CNVs to breast cancer risk. Furthermore, the huge number of cases and controls available for future CNV association studies will provide sufficient power to evaluate many CNVs that occur at low frequency. A major limitation with using these array data is the inability to genotype highly repetitive copy number-variable regions. More than 1000 regions across the human genome have been found overlapping CNVs with three or more segregating alleles [72]. Non-array-based technologies that can resolve multicopy integer states, such as qPCR, Nanostring and massively parallel sequencing, will therefore be necessary to determine the clinical significance of these multiallelic variants in breast cancer and other human diseases.

### 5.2. Inherited and de novo Rare CNVs and Breast Cancer Risk

At least seven array-based studies have reported lists of rare CNVs overlapping genes that may contribute towards the development breast cancer [8,73,74]. Despite a number of candidate susceptibility genes being proposed there has been a notable lack of concordance between these studies. More than 120 genes overlapping rare genomic deletions or duplications have been found exclusively or at a greater frequency in familial breast cancer cases; however, none have been replicated between studies (Supplementary Table S1). Such a finding is not surprising as many individuals carry rare or private CNVs regardless of their disease status [2,75]. Furthermore, four of these studies used SNP-based arrays which are known to generate signal-to-noise ratios that are much lower than array-CGH platforms and are therefore more prone to false CNV calls [58]. It remains unclear whether future large-scale studies will provide the reproducible evidence needed to implicate these rare CNVs as breast cancer risk variants and to overcome the issue of false discovery.

Growing evidence suggests that the frequency and size of constitutional CNVs are significantly increased in breast cancer-affected individuals [73,74,76]. Studies have assessed the global burden of deletions and duplications in cases and controls by measuring: (1) the number of CNVs per sample; (2) the number CNVs overlapping genes (and *vice versa*) per sample; (3) the average length of CNVs per sample; and (4) the total number of base pairs affected by CNVs per sample. Although studies have revealed a common trend of increased CNV burden in breast cancer cases, the trend appears to be strongest when assessing CNVs that overlap gene regions [73,74]. Evaluating such genes further by pathway analysis suggests two networks centred on factors known, TP53 and β-estradial [73], may be important in breast cancer risk and development; however, these findings are yet to be reproduced. The feature of “CNV burden” has also been observed in the genome of patients with other cancers, suggesting that an uncharacterised subset of these variants may be causal [77,78,79,80]. Further studies are needed to identify recurring variants at shared loci.

### 5.3. Is There a Relationship between Germline CNVs and Breast Tumourigenesis

A characteristic of sporadic and familial breast tumours is genomic instability, resulting from either inherited mutations in genes that control genome integrity, or mutations that are acquired in somatic cells during development. Breast tumour cells in carriers of the *APOBEC3A-APOBEC3B* germline deletion show a greater number of C>T transitions than in non-carriers [81], thereby highlighting the importance of this common CNV in breast cancer development. It has previously been proposed that germline CNVs may also contribute to somatically acquired chromosome changes in tumours. Previous studies of Li-Fraumeni Syndrome (LFS) tumours [80] and of colon cancer-affected individuals [82] suggested that constitutional CNVs may act as a foundation on which chromosome copy number aberrations develop in tumour cells. These findings suggested a direct relationship between constitutional genomic variation and tumour genome evolution. The notion that inherited CNVs may influence the occurrence of somatically acquired copy number changes during breast cancer progression has not only prognostic significance, but also important consequences for early decisions relating to clinical management. Subsequent analyses of constitutional and tumour-specific CNVs in matched breast tumour and normal tissue using data from the Illumina Human CNV370 duo beadarray provided evidence that the location of copy number aberrations in tumour cells do not associate with constitutional CNVs [83]. However, the SNP arrays used in these studies had a relatively low number of probes and therefore poor spatial resolution for detecting CNVs and defining the variant boundaries. To determine the relationship between inherited genomic variation and genome evolution in breast cancer, sequencing-based studies are necessary to ensure accurate mapping of CNV breakpoints.

## 6. Conclusion

Genotyping constitutional CNVs using low- and high-resolution SNP arrays has served as the primary screening method for identifying potential genetic markers associated with breast cancer risk. Despite the large amount of SNP array data available from breast cancer studies, the contribution of inherited copy number variation to breast cancer risk remains relatively understudied. A variety of algorithms have been generated and matched to these datasets for predicting copy number-affected regions throughout the genome. Applying such algorithms may reveal new common and rare variants that contribute to breast cancer risk. However, initial analyses suggest array-based CNV data may be unreliable without further validation using ancillary technologies, such as qPCR, Nanostring, and MLPA. Moreover, the current and future use of new higher resolution technologies, including next-generation sequencing, will be critical for characterising CNV breakpoints, to better interpret their potential impact on breast cancer risk.

## Supplementary Files

Supplementary File 1
